# Facilitators and barriers to the practice of intermittent auscultation fetal monitoring in UK maternity services: a qualitative study using the Consolidated Framework for Implementation Research (CFIR)

**DOI:** 10.1136/bmjopen-2025-115855

**Published:** 2026-04-24

**Authors:** Jennifer MacLellan, Mo Ade, Megan Douthwaite, Bev Fitzsimons, Karen Joash, Sumayya Mulla, Julia Sanders, Sara Kenyon, Catherine J Pope, Rachel Rowe

**Affiliations:** 1 National Perinatal Epidemiology Unit, University of Oxford, Oxford, UK; 2 National Maternity Voices, Southeast England, UK; 3 Formerly of The Point of Care Foundation, London, UK; 4 Obstetrics and Gynaecology, Imperial College Healthcare NHS Trust, London, England, UK; 5 Expert by experience contributor, North England, UK; 6 School of Healthcare Sciences, Cardiff University, Cardiff, UK; 7 Public Health, Epidemiology and Biostatistics, University of Birmingham, Birmingham, UK; 8 Nuffield Department of Primary Care Health Sciences, University of Oxford, Oxford, Oxfordshire, UK; 9 NIHR Policy Research Unit in Maternal and Neonatal Health and Care, University of Oxford, Oxford, UK

**Keywords:** Midwifery, Implementation Science, Quality in health care, QUALITATIVE RESEARCH

## Abstract

**Abstract:**

**Objectives:**

To explore barriers and facilitators to midwifery practice of intermittent auscultation according to national guidance in the UK.

**Design:**

Multisite ethnographic study using observations of practice, semistructured interviews and informal conversations. Framework analysis using the Consolidated Framework for Implementation Research (CFIR).

**Setting:**

11 maternity units across seven NHS maternity services in England and Wales in 2024.

**Participants:**

Midwives and other maternity care professionals involved in fetal monitoring during labour.

**Intervention:**

‘Intermittent auscultation’ (IA), or listening to the fetal heart rate at regular intervals, to monitor fetal well-being during active labour.

**Outcome measures:**

Not applicable.

**Results:**

IA monitoring was frequently observed to be marginalised due to national and local pressures. IA is a complex skill that requires expertise and practice to develop and maintain. However, lack of a robust evidence base for IA methods is a further barrier to implementation. The study uncovered examples of facilitators that include: leadership engagement, access to knowledge and information supported in mentorship programmes and peer support models. These features created micro-environments where IA was valued, supported and integrated into care.

**Conclusions:**

Our study highlights the significant impact of multilevel factors on the implementation of IA within UK maternity care. However, when organisational readiness, strong leadership engagement and supportive conditions are present, IA can be delivered in accordance with guidance. These findings underscore the need to align policy, infrastructure and organisational culture to sustain evidence-based, woman-centred practices such as IA.

**STRENGTHS AND LIMITATIONS OF THIS STUDY**

- The ethnographic approach provides unique insights into the how and why of ‘intermittent auscultation’ (IA) implementation.
- Analysis using an implementation framework provides clear direction of where to target resources for implementation support.
- A sample size of 11 maternity units may impact transferability of findings.
- Data were collected by one researcher; however, perspectives of the whole research team were incorporated through a group analysis approach, strengthening the trustworthiness of the analysis.
- There were limited opportunities to observe midwives using IA in labour due to low numbers of women experiencing IA in their labour during the observation period.

## Background

 In the UK and around the world, midwives use ‘intermittent auscultation’ (IA), or listening to the fetal heart rate at regular intervals, to monitor fetal well-being during active labour. IA, conducted for a specified duration, at a specified time in relation to uterine contractions, can identify changes in the fetal heart rate and pattern that may indicate inadequate oxygen supply to the baby.^1^ Additional care or intervention, for example, monitoring the fetal heart rate using continuous electronic fetal monitoring (CEFM), or expediting birth, can then be provided if indicated. IA was first proposed in 1821 as a method of fetal wellbeing assessment, with the fetal stethoscope developed in the 1920s along with a recommended programme of auscultation.^2^ It is the recommended method of fetal monitoring during labour for healthy women with uncomplicated pregnancies in the UK and internationally and is associated with fewer interventions during labour and birth than CEFM, with similar perinatal outcomes.^3^ Current guidance for England (and across the UK) recommends that IA is performed immediately after a contraction for a full minute using a handheld fetoscope (pinard) or Doppler ultrasound device. This should be done every 15 min in the first stage of labour, increasing frequency to every 5 min in the second (pushing) stage.^4^ There is no guidance about how the fetal heart rate should be counted, with different approaches including counting for the full minute or counting in blocks of 15 s for 90 s and taking an average.^5^ Most international guidance about IA is similar to that used in the UK, with some variation, particularly around frequency.^6 7^

Variation in midwives’ practice of IA internationally is well-documented.^5 8^ Successive UK enquiries have highlighted issues with IA practice contributing to avoidable harm for babies.^9^,^12^ The physical environment, workload demands and the intensity of monitoring over a long period of time have been suggested as possible explanations for observed differences between IA guidance and IA in practice.^12^ Structural issues such as staffing shortages, poor Information Technology infrastructure, inconsistent access to functioning Dopplers or inappropriately equipped birth spaces can also undermine midwives’ ability to practise IA in line with guidance.^13^ Inadequate risk assessment for IA monitoring, escalation when abnormalities are detected and poor recognition of transition between stages of labour have been highlighted as areas of concern in the safe practice of IA monitoring.^14^ Risk assessment and escalation toolkits are being trialled and implemented across theUK National Health Service (NHS),^15^ with increased frequency of IA audit and improved training also recommended.^16 17^ Against this backdrop, there is little research evidence,^7 12^ and a pressing need to examine the organisational and practice context to inform how to best support midwives to provide high-quality IA.

In this study, nested in a larger implementation study (Listen2Baby), we explore midwifery practice of IA to illuminate the barriers to and facilitators of following national guidance. In Listen2Baby, these findings were used to inform the co-design of a toolkit of resources to support IA practice. This co-design process will be reported elsewhere.

We use the term ‘woman’ throughout. This term should be taken to include people who do not identify as women but are pregnant or have given birth.

## Method

### Study design

The research question for this study was:

What are the facilitators and challenges for midwives in practising intermittent auscultation monitoring according to national guidance in England?

To answer this question, we conducted a multisite ethnographic study comprising observation of midwives looking after women in labour, semistructured interviews and informal conversations with midwives, student midwives, obstetricians and maternity support workers.

### Theoretical approach

We used the Consolidated Framework for Implementation Research (CFIR) as an overarching conceptual framework for the Listen2Baby project. The CFIR guides the exploration of facilitators and barriers to understand why an intervention may or may not work in a particular setting.^18^ It has been successfully used to evaluate the implementation of complex healthcare interventions.^19^,^21^ The CFIR is intended to be flexible and can be tailored to particular innovations and contexts; not all constructs are expected to be relevant to all innovations.^18^ In this study, we used the CFIR domains and their underlying constructs to guide data collection and analysis, and to structure the presentation of our findings about the principal drivers of and barriers to implementation of IA in practice. These findings informed the co-design of solutions which formed the basis of the Toolkit (to be reported elsewhere)^22^ . Several of the CFIR constructs were relevant in some way in our study; those domains and constructs most strongly expressed in the data are shown in table 1.

**Table 1:** CFIR domains and how they were applied in this study, with constructs and those expressed in our data (in bold)

CFIR domain and what it means in our study	Constructs (bold denotes application to IA practice)
**Innovation** The ‘thing’ being implemented: Intermittent auscultation according to guidance	**Source** **Evidence base** **Relative advantage** **Adaptability** Trialability **Complexity** **Design** **Cost**
**Outer setting** National and regional factors impacting implementation: The policy context surrounding fetal surveillance, maternity enquiries, litigation cases and opinion reported through the media.	**Critical incidents** Local attitudes **Local conditions** Partnerships and connections **Policies and laws** **Financing** **External pressure**: Societal, Market, Performance measurement
**Inner setting** Internal/institutional factors impacting implementation: The maternity unit or community setting where birthing women are monitored using IA*.*	**Structural characteristics** Relational connections (at a structural level) Communications **Culture** Tension for change Compatibility Relative priority Incentive systems **Mission alignment** **Available resources** **Access to knowledge and information**
**Individuals** The midwives and birthing women impacted by the practice of IA.	High-level, mid-level, **opinion leaders:** Need, capability, opportunity, motivation **Implementation facilitators, leads, team members**, Need, capability, opportunity, motivation **Innovation deliverers** and recipients Need, **capability, opportunity, motivation**
**Implementation process** Strategies and activities used to implement the innovation: Processes in place in the practice context to support the practice of IA.	**Teaming** **Assessing needs** **Assessing context** Tailoring strategies Engaging Doing Reflecting and evaluating Adapting

CFIR, Consolidated Framework for Implementation Research; IA, 'intermittent auscultation'.

### Participants and sampling

The ethnography was conducted in 11 maternity units across seven NHS maternity services (referred to below as ‘sites’) in England and Wales. Site selection was informed by mapping characteristics of NHS maternity services, including geographic location, type and configuration of unit(s), annual birth numbers and population-level indicators of ethnic diversity and deprivation. Eight sites were initially selected to represent maximum variation but, due to governance delays and the achievement of data saturation, seven sites participated.

We aimed to recruit maternity practitioners (midwives, obstetricians, student midwives and maternity support workers) in each site for interview, informal conversation or observation of practice. Posters displayed in maternity units informed staff, women and their families about the research. Staff also received prior notification about the study and presence of the researcher at site by email from senior staff and through staff meetings. Participants who were eligible and interested in being interviewed received an information sheet about the study and were invited to contact the researcher. Midwives practising IA during labour were approached by the senior midwife on duty and asked permission for their practice to be observed. If they agreed, they then sought permission from the birthing woman and her family for the researcher to be present in the room. The permission of the woman and the presence of the researcher were recorded in the woman’s notes. An information sheet about the study, including the researcher’s contact details, was given to the family.

We purposively sampled for diverse experiences of and perspectives on IA, including from obstetricians and maternity support workers, to give a broad holistic view of the context in which IA is practised. We collected data from participants we spoke to or observed about how long they had been qualified, and clinical experience, to ensure this diversity.

### Data collection

Data collection took place between December 2023 and December 2024, during day, night and weekend shifts. Templates for observation were developed, informed by Spradley’s nine dimensions of descriptive observation. ^23^Topic guides for single-episode interviews were informed by the CFIR and included open questions about what supported or hindered IA practice. Interview findings were sense-checked during informal conversations and new lines of enquiry uncovered. All interviews were conducted either online or in person following informed consent, by an experienced qualitative maternity researcher (JM), who also conducted the observations. Audio recordings were transcribed verbatim, checked against the original recording and loaded into the data analysis software, NVivo 14.^24^ Field notes were used to record informal conversations, observations of IA practice and activity in non-clinical areas. These were typed up at the end of the shift and stored in a password-protected document, with original notes destroyed securely.

### Data analysis

We analysed the data thematically as data collection progressed. The researcher coded the transcripts and observation field notes inductively and discussed the coding frame and results in regular analysis meetings with the wider multidisciplinary research team which included two lived experience members. Analysis progressed to focused coding and development of core categories. Data collection continued until data saturation was reached.^25^ We mapped core categories to the CFIR framework, enabling us to systematically identify barriers to and enablers of IA practice across the different maternity care settings. This approach allowed us to explore not only what influenced IA practice, but also how and why these factors operated within specific organisational, professional and practice contexts.

### Patient and Public Involvement

The core research team comprised two lived experience members who were involved in the team data analysis meetings during the ethnography, critiquing the data and suggesting areas that required further interrogation. As part of the toolkit co-design process, these findings were sense-checked in a collaborative workshop involving maternity professionals and 12 women with lived experience of IA in their labour who had participated in the women’s interviews that informed the Listen2Baby study.^26^ While some findings were considered to be quite technical about monitoring practice, feedback from women with lived experience about the offer of IA, talking with women and the environment of IA practice confirmed what we had observed.

### Findings

We collected data from 11 maternity units within seven NHS maternity services (sites) (table 2).

**Table 2:** Characteristics of study sites

Site	Size (births/year)	Location	Configuration	Deprivation*	Ethnic diversity†
Azure (Comprising 3 units: Central maternity unit OU+AMU, district general hospital with OU+AMU and FMU)	>6000	Urban	OU/AMU & FMU‡	30%–40%	40%–50%
Indigo	3–6000	Urban	OU only	10%–20%	<5%
Lime	3–6000	Urban	OU/AMU	30%–40%	10%–20%
Magenta (Comprising 2 district general hospitals with OU+AMU)	<3000	Urban/rural	OU/AMU	20%–30%	<5%
Teal	>6000	Urban	OU/AMU	>50%	40%–50%
Yellow	3–6000	Urban	OU/AMU	10%–20%	20%–30%
White (Comprising 2 units: 1 district general with OU+AMU and 1 FMU)	3–6000	Urban/rural	OU/AMU & FMU	<10%	<5%

* % of all births that fall within top quintile for child poverty.^28^

† % of all births to Black or Asian women.^28^

‡ OU (obstetric unit): midwife-led care with obstetric, anaesthetic and neonatal services on site. AMU (alongside midwifery unit): midwife-led care located on the same site as an OU. FMU (freestanding midwifery unit): midwife-led care on a separate site from an OU; requiring transfer by ambulance or car to the OU if medical or neonatal care is needed.

AMU, alongside midwifery unit; FMU, freestanding midwifery unit; OU, obstetric unit.

Study sites included those with Care Quality Commission (CQC) ratings of ‘inadequate’, ‘requires improvement’ and ‘good’ at their most recent inspection.

Data consisted of 313 hours of observation (average 45 hours in each site), 98 informal conversations, 37 recorded interviews (average 35 min 24 s), and 13 observations of midwives using IA during labour. Characteristics of participants are presented in table 3.

**Table 3:** Characteristics of participants

Profession		Interview	Informal conversation
	Midwife	33	83
	Student midwife	3	12
	Obstetrician	1	2
	Maternity support worker	0	1
Years qualified	Student	3	12
	<1	2	4
	1–5	7	22
	6–10	7	24
	11–15	4	9
	16–20	5	13
	>20	8	13
Current area of work	Obstetric unit	6	20
	Midwifery unit	14	56
	Community	12	5
	Other (consultant midwife, fetal monitoring midwife)	5	17

### The innovation: IA according to guidance

In this study, the innovation, the ‘thing’ being implemented, was IA according to National Institute of Clinical Excellence guidance. We used the CFIR constructs of innovation source, evidence base, relative advantage, adaptability, complexity, design and cost to build a picture of the innovation in the context of practice.

The source of IA practice lies firmly within the midwifery profession and midwifery-led care. IA was seen by participants as a core midwifery skill:

that’s your bread and butter, isn’t it? You know, going back to your Pinard, then you’re IA. MagentaStar05, OU, Midwife, 18 years qualified

Its use in midwifery-led care positions it as a low-tech activity, and as a ‘step-down’ option from the more technological and highly regarded CEFM.

The lack of respect is from other midwives, saying there is nothing to do for low risk women, it is not respected. Lime, Informal Conversation, AMU, Midwife, 22 years qualified

IA is not a new technology but requires expertise. This expertise integrates careful listening and counting of the fetal heart, with little or no permanent record of that sound; clinical reasoning; experience; evidence-based knowledge; and the clinical context of the labouring woman. The rapid pattern recognition of expert practice acts below conscious awareness, making knowledge automatic but often simultaneously inaccessible to verbal explanation, rendering the skill invisible.^27^

While apparently technically straightforward, IA was observed to be a complex skill, requiring clinical judgement and interpretation, focused concentration and regular practice to achieve and maintain competence—challenges that were intensified in busy or understaffed environments (Observation field notes-Indigo). Some participants viewed it as more time-intensive than CEFM. IA was largely absent from obstetric unit (OU) practice in our sites. Risk assessments during labour check if IA is still an appropriate method of monitoring but were not used to question the appropriate or continued use of CEFM, suggesting IA is not given equal status as a practice (Observation field notes-Azure). IA cases were not presented or discussed in the weekly fetal monitoring educational meetings (Observation field notes-All sites). Antenatally, documentation suggested that IA was rarely discussed with women as a monitoring option; instead, conversations would focus on birth setting, which by default dictated the method of monitoring (IA in the midwifery-led unit, CEFM in the OU). Job descriptions for fetal surveillance midwife roles had little or no mention of IA, in contrast to CEFM (Teal field notes), and there was little evidence of training or other resources to support IA.

I think the Doppler comes second place to a CTG… the perception is where all the high-risk and really important stuff matters is on the delivery suite. White04, Midwife, >20 years qualified

IA was observed to be, and described as, adaptable to the movement and position of the woman and the progress of her labour (Observation field notes-All sites). It was practised with increased frequency or altered timing to give additional information to the clinical picture as required. it’s quite beautiful. A woman can move around, she’s no trouble. She is, she can do whatever she wants to do. Indigo04, OU, Midwife, 18 years qualified

so you are listening, and I guess you use your judgement, don’t you? If you think that you can hear anything and then you just listen again, or you extend it, so it’s not you’re tied to a minute. MagentaStar04, Community, Midwife, 10 years qualified

### The outer setting: national and regional factors influencing IA practice

The CFIR outer setting refers to the wider context around the inner setting; in this study, this included the policy context around fetal surveillance, maternity enquiries, litigation cases and opinion reported through the media. Our analysis identified critical incidents, local conditions, policies and litigation, financing and external pressures impacted on IA practice.

Clinicians are required to take reasonable care to ensure that the woman is aware of any material risks involved in any recommended treatment, and of any reasonable alternative or variant treatments.^28^ A focus on risk was evident in IA practice, driving the documenting of conversations and offers of care.

I mean it’s one of the main challenges of the job, isn’t it, to try and… we’re so… it’s so risk focused on that documentation, is such a huge part, and that’s the thing that you tend to get hung up on a lot. MagentaStar04, Community, Midwife, 10 years qualified

Midwives described how media coverage of UK maternity enquiries, often explicitly critical of midwives and/or midwifery, and suspension of the ‘normal birth campaign’ by the Royal College of Midwives had shaped a climate of defensiveness in midwifery practice.^29^ Midwives expressed reluctance to champion IA due to its association with ‘normal birth’.

These ‘outer setting’ pressures led to IA being regarded locally as a marginalised practice rather than a mainstream option, positioning CEFM as the default option. Consequently, one participant described IA as a ‘lost art’ (Azure02, AMU midwife, 20 years qualified).

I think it’s because of the way that we practice, which is defensive, it is defensive, and I think just knowing you’ve got the backup of that trace sometimes. Indigo05, OU, Midwife, 2 years qualified

Midwives working in maternity units rated as ‘inadequate’ or ‘requires improvement’ by the Care Quality Commission (48% of all maternity services inspected in 2024)^29^ reported a heavy burden of audit, continuous quality improvement projects and weekly reporting, all of which added to existing clinical workloads.

so we were downgraded on the last CQC to inadequate, or whatever it is, and then they came back, and then they issued this section 37, which basically, there’s five workstreams that are going on where the trust need to provide evidence that these things are happening. But as a result, we are overwhelmed now with audit and proving your care that we’re providing, rather than just providing the care. White03, AMU, Midwife, 16 years qualified

These demands left staff feeling emotionally and physically depleted, reducing their capacity to provide the kind of relational, midwife-led care in which IA is typically embedded, and this encouraged routine use of CEFM (Observation field notes-Azure & White).

National guidelines were interpreted differently across sites, often dependent on local leadership. As a result, women with similar clinical profiles, but in different units, could receive different care. In some sites, participants reported that medical staff overseeing induction of labour and midwives in triage were unfamiliar with IA eligibility and often recommended CEFM ‘as per protocol’ (Informal conversation, OU, midwife, MagentaStar). This created inequities in access to and use of IA.

It’s quite easy to find risk factors, and to be fair, the decision is made from triage and (I) would not question it. Indigo, Informal conversation, OU, Midwife, 1 year qualified

Participants widely reported a decline in the frequency of IA use and the confidence of midwives and students in practising it. This was attributed to: a lack of nationally agreed training that led to local practice variation (not all sites taught fetal physiology as applied to IA monitoring, resulting in different warning signs prompting escalation and questions about its credibility); increased complexity within the birthing population; and workforce issues (including moving midwives from the alongside midwifery unit (AMU) to the OU) that left fewer staff available to confidently use IA (Observation field notes-Indigo & Teal),

10 years ago IA wasn’t in the annual update training because it was the norm. Now there are students and Band 5s coming through with little experience of IA. Teal, Informal conversation, AMU, Midwife, 15 years qualified

Closure or removal of night-time staffing in a freestanding midwifery unit (FMU) reduced the number of births there and impacted midwives’ opportunity to maintain their IA skills. Similarly, midwives undertaking the newborn examination (NIPE), or specialist roles such as bereavement midwives, were described as unintentionally diverting experienced staff away from frontline labour care:

Labour care is now given by junior midwives who have not been trained on normal birth. Indigo, Informal conversation, Midwife, 12 years qualified

In contrast, units with strong, integrated multidisciplinary leadership created systems that actively supported IA practice (as discussed in the inner setting, below).

### The inner setting: in which the innovation is implemented

In the context of this study, the ‘inner setting’ was the maternity unit and the care episode between midwife and woman in the birthing room. The principal constructs reflected in our data were structural constraints (physical, information technology and work infrastructure), culture, mission alignment, available resources and access to knowledge and information.

IA requires midwives to listen to and count the fetal heart for a specified duration. Doppler ultrasound devices, that were most widely used, produce an audible fetal heart sound which needs to be counted by the midwife, with some devices also showing the fetal heart rate on a screen. The birthing environment was not always optimised for IA practice to enable midwives to see a clock; midwives were observed using mobile phone lights, stopwatches on their phone and repositioning clocks (Observation field notes-All sites).

Sometimes I do have my phone light on upside down in the corner… ’cause I don’t wanna interrupt the woman’s space either: she’s in the zone. Lime01, Community Midwife, 32 years qualified

Doppler devices were reported to be available in every birth room, but in practice, they were often missing, awaiting repair or rendered unusable by over-complicated battery replacement procedures (Observation field notes-All sites). Clinically based midwives had little input in the choice of Dopplers purchased. In addition, in four of the seven sites, digital documentation posed an additional barrier to IA monitoring. Weak Wi-Fi in birth rooms, or a lack of laptops/tablets, left midwives documenting on paper and uploading data later, sometimes long after care had been delivered (Observation field notes-Indigo, Teal, White, Yellow). Midwives preferred the flexibility of handheld tablets where they were available.

There are sometimes not enough laptops to go around so you have to come in here (office) which might be okay in 1st stage but not in 2nd so you are scribbling on a piece of paper. I much prefer paper than you can have it with you and be contemporaneous, or at least timely. Teal, Informal conversation, Midwife, 9 years qualified

In one unit, space constraints meant digital documentation had to occur outside the birth room entirely (Observation field notes-Teal). Participants highlighted frustrations with digital systems that automatically logged users out after brief idle periods, creating delays and discouraging real-time data entry:

She has to write everything in retrospect… misses her break or goes off shift late… is pulled onto another job and then is delayed in her retrospective write-up. Teal, Informal Conversation, AMU, Midwife, 3 years qualified

Participants reported that it was not always obvious in digital records whether the woman was eligible for IA because of how risk status was displayed. Locating relevant history often required cross-checking fragmented records, particularly for women who had previously received care in other regions or systems. Training on digital systems was reported to focus on navigation rather than practicalities during labour. In two sites, IA was primarily documented on a paper partogram (a chart showing a visual overview of fetal heart rate, maternal vital signs and contractions over time), with narrative entries in the notes reserved for abnormalities. This streamlined approach to documentation was appreciated by midwives as it allowed them to spend more time on direct care.

Midwifery units were described by participants as being staffed independently, but in practice, OUs took priority in staffing. Midwives were frequently ‘escalated’ from the midwifery unit to the OU (Observation field notes-Azure, Lime, Teal, White):

Staffing is often an issue, Birth Centre (AMU) escalated up to Delivery Suite (OU) but Delivery Suite (OU) will refuse to come down to Birth Centre (AMU). Teal, Informal Conversation, AMU, Midwife, 3 years

This led to temporary closures of AMUs and FMUs, restricting options for women who had planned birth in these settings, resulting in fewer opportunities for student and staff training, and placing further strain on the midwives striving to maintain this as an option:

In her band 5 rotation ‘preceptorship year’ she only spent one day on the AMU as she was always escalated (moved) out to other areas. Teal, Informal conversation, AMU, Midwife, 2 years qualified

Support from OU staff for IA practice, including providing support on the AMU or practising IA on the OU, was rare. Conversely, in two units where a senior AMU coordinating midwife was always on shift, IA was reliably delivered. This occurred as a result of local leadership advocating for care of eligible women in the AMU, with oversight of the co-ordinator to support more junior staff. In some units, women who were booked for midwifery-led care were admitted directly to the AMU for assessment, reducing the chance of default CEFM in an obstetric triage unit (Observation field note-Lime, Magenta, White). Another local strategy that supported IA practice was multidisciplinary review of induction of labour lists, and in one site, a consultant obstetrician routinely questioned the use of CEFM in women without risk factors, reinforcing reflection and recognition of IA as a safe and appropriate option.

We observed different approaches to teaching and training in IA across our sites. In three maternity units, senior midwives taught fetal physiology applied to IA in the undergraduate midwifery curriculum and during ‘in-house’ training (Observation field note-Azure, Lime, White). This ensured students were familiar with IA principles and practice before arriving in the clinical area and encouraged staff to remain up to date with IA guidelines. Ongoing support was beneficial.

So I think me personally it takes a lot to get my head around it (fetal physiology), so I think once a year isn’t enough ’cause I feel like: ‘oh, yeah, I got some of that,’ and I know come next month I’ll be like: ‘oh, I forgot about that.’ Yellow02, AMU, Midwife 6 years qualified

Not all sites taught fetal physiology as applied to IA monitoring, but in those that did, midwives described understanding better what they were listening to and that it was more accurate. This made them feel more confident talking with medical staff and on transfer of the woman to the OU, as they could back up their observations with a physiological explanation.

I feel like I’m looking at the physiology of the baby, I feel like I’m listening to physiology; whereas (without knowledge of physiology), I feel like I’m just doing numbers, it doesn’t feel the same. Yellow01, AMU, Midwife, 9 years qualified

actually since I’ve been doing it more, I do think it’s more accurate, and I think that it makes you think about the bigger picture a bit more. Lime06, OU, Midwife, 10 years qualified

We observed different approaches to fetal heart rate counting. Without evidence, different sites either enforced a particular method or left midwives free to choose. Where a counting method was mandated, those midwives who did not feel confident using it described feeling their knowledge and ability to monitor fetal well-being during labour was questioned, which in turn undermined their clinical confidence;

now we’ve got this IIA (block counting), you do a module – which we all fail and then what, how do you do that in practice? Are we all doing it right? MagentaStar, Informal conversation, Community, Midwife

We observed fetal monitoring midwives providing direct clinical support in IA to colleagues in two sites. This leadership presence fostered an open and confident team culture.

And that’s how we work here, we give the support; if people need extra support, we will give it. I was supposed to be working with (colleague) this morning, I will come on shift, and if she needs any help, I will be her support mechanism. Azure02, AMU Midwife, >20 years qualified

Similar support for IA practice was seen in two sites where a ‘peer review’ support model was used, whereby a second midwife conducted a regular holistic review of the woman, the baby including IA monitoring and the attending midwife’s well-being. This was in contrast to a ‘fresh ears’ regular check of the fetal heart alone by a second midwife, which some described as their practice being ‘checked’.

### The individuals: the roles and characteristics of individuals involved

This domain focuses on the characteristics of the individuals practising IA. The principal constructs identified in our data were motivation, opportunity and capability of implementation deliverers. Characteristics of leaders at different levels of the organisation and opportunities for IA practice have been covered under the inner setting.

Participants who saw IA as part of their professional identity described a deep personal incentive for offering IA in alignment with guidance. One participant shared:

I do have ladies and (the monitoring) it’s intermittent auscultation, it is really free and does end up feeling really lovely in the end… it feels like real midwife skills when you know you deliver that low-risk baby and you’ve used intermittent auscultation… it does feel amazing. Indigo05, OU, Midwife, 2 years qualified

Supportive colleague relationships mitigated against staffing constraints, as participants were observed to stay beyond the end of their shift, miss breaks, cover staffing gaps and change clinical areas to support their colleagues. This goodwill and flexibility among individuals passionate about physiological birth was a key enabler of IA practice.

In some sites, the responsibility for offering IA monitoring to eligible women appeared to rest with individual midwives rather than being embedded within leadership or service support structures. Some participants described how they did not understand how IA works or that it takes too much effort; they preferred the ‘objective’, easier continuous reading produced by CEFM. This felt more professionally ‘safe’ for the midwife, as they perceived it as more able to withstand legal challenge, but some midwives also implied that CEFM was ‘safer’ for the baby too. This attitude could impact the risk assessment for IA monitoring. Conversations were framed as ‘CTG is safer’ and this meant that a return to IA monitoring after a reassuring CTG was uncommon.

Sometimes I feel it is done for the convenience of the midwife because it is easier and it’s how its phrased to convince the woman it’s the best thing for her. Lime, Informal Conversation, Third year student midwife

you know, straight onto a CTG (CEFM) ’cause people feel more confident ’cause they can see what’s happening. People don’t like not knowing. Azure 02, Midwife, >20 years qualified

Midwives’ confidence in using IA was central to IA practice. Midwives who had significant experience of supporting women using IA monitoring described feeling confident in their ability to perform and interpret auscultation reliably.

I do put a Doppler on someone and I think it’s just experience, so you put a Doppler on, you’re like, ‘ouu, that doesn’t sound right,’ we listen for longer, that type of thing; it’s more experience than training. Magenta01, Community midwife, 14 years qualified

Some participants did not trust IA in comparison to CEFM as it only gave them a ‘snapshot’ and they expressed concern about what they might be missing in between listening episodes. Many midwives (principally those who worked in OU or triage settings) and medical staff in our study described not believing IA to be a robust monitoring tool, perhaps because of their lack of understanding of IA practice.

The Consultant looks for reasons to put on CTG (continuous electronic monitoring) and shudder when they hear IA, you have to advocate for it and stand your ground. Indigo, Informal Conversation, OU, midwife, 2 years qualified

lots of labour ward MWs won’t come down to the B/C. If they do, they will put the woman on a CTG as they don’t trust IA. Azure, Informal conversation, midwife, 9 years qualified

### The implementation process

This domain refers to the activities and strategies used to implement the innovation in practice. Since IA is already an embedded midwifery practice, we used this to consider what supports IA practice, or not, using the constructs teaming, assessing needs and context.

Teaming refers to the degree to which individuals work together to implement the innovation. Within our data, leadership played a vital role in the implementation of IA practice. This was shown, for example, by the consultant midwife checking induction lists and pro-actively offering IA to eligible women; fetal monitoring midwives being in clinical practice supporting staff in IA practice; senior midwives co-ordinating and supporting IA practice on the unit; escalation of serious IA concerns straight to theatre rather than to OU for a ‘check CTG’; and multidisciplinary support of IA as an appropriate approach to fetal monitoring in labour.

And I think having consultant input, she’s very involved. So having her there really helps because I think showing a midwife and an obstetrician together in… not just mandatory, but weekly, and I think that in itself shows that you have to work together. Yellow 01, midwife, 9 years qualified

This multidisciplinary respect of midwife expertise and decision-making, with the support of colleagues and leadership, was observed to support IA practice according to guidance.

Midwives working together was particularly apparent in the second stage of labour, when guidance recommends the monitoring of the fetal heart every 5 min. This was observed and described as logistically challenging as the midwife must support the woman and partner, provide perineal protection, open packs, count swabs, check and draw up oxytocin, continue maternal observations, monitor and document the Fetal Heart every 5 min, and document progress, all while gloved in preparation for the birth.

it’s the practicalities of that, finding the timing, alongside all your documentation and everything, is sometimes very difficult to keep up with. Indigo06, Community, midwife, 4 years qualified

it’s no longer in the guideline to have a second (midwife), and there isn’t enough staff anyway, you might have an maternity support worker or student but often you are alone and you just get on with it. Teal, Informal conversation, AMU, midwife

Midwives are expected to call a second staff member for the birth to provide care to the baby, but we observed midwives working together earlier in the second stage, where staffing and the work environment allowed, to support each other in the practice and documentation of IA.

Just buzz and then they will come in and they then tend to take over the note side of things so that I can just deliver,…’cause it’s hard trying to listen, protect the perinium, deliver the head, and look at what time it is. Yellow02, AMU, Midwife, 5 years qualified

In two sites, midwives who did not feel confident in IA were supported in their practice through a programme of shadowed practice and mentoring.

## Discussion

This study explored the context of IA practice in the UK. We used the CFIR conceptual framework to identify what works, where and why, highlighting the complex and often constraining influence of multilevel factors that act to undermine IA as the recommended method of intrapartum fetal monitoring for women with low-risk pregnancies.

IA monitoring is endorsed in national guidelines for uncomplicated births, but was frequently observed to be marginalised due to national and local pressures. The lack of a robust evidence base for IA methods is a further barrier to implementation; there is little high-quality evidence on optimal devices (Pinard vs Doppler), counting methods or recording strategies.^6^ When clinical enquiries arise, IA practice is difficult to defend among clinical colleagues (even though CEFM itself is not supported by strong outcome data).^3^ Implementation science literature shows that weak evidence can erode practice fidelity and sustainability, particularly when alternatives appear more measurable or defensible.^20^ From an implementation perspective, CEFM benefits from the ‘visibility’ of its continuous trace, that reinforces credibility and is easily integrated into digital workflows and audit trails.^30^ This aligns with implementation frameworks that highlight how perceived relative advantage and observability influence uptake.^31^

IA, by contrast, is embodied and relational. It is a complex skill that requires expertise and practice to develop and maintain. Counting and interpretation leave no visible record other than notes made by midwives—in contrast with the digital CEFM recorded trace. IA is a midwifery skill, practised only by midwives—in contrast with CEFM which is a medical technology used by midwives and other staff. In practice, midwives must balance attentive listening with competing tasks, which constrains timely and detailed recording.^32^ Clinical enquiries frequently highlight poor IA documentation as a contributory factor in the review of adverse outcomes.^14 33^ This makes midwives’ documentation in labour vital to ensure accurate data against which IA practice can be measured and supported.^33 34^ Documentation gaps reduce both confidence and defensibility, reinforcing reliance on CEFM, which ‘documents itself’.^35^

Workforce issues provide further implementation challenges. Where CEFM is so commonly used, midwives have fewer opportunities to practise IA, which can erode confidence and maintenance of their skills. Midwifery students may graduate without achieving competence in IA if clinical placements rarely use it^36^ and over time, this deskills the workforce who become less able to deliver IA reliably. The absence of standardised training across maternity services further undermines implementation.^37^ Teaching of IA in university and the clinical area varies across the country. This results in inconsistent technique, interpretation and documentation.^32^ Without agreed training standards or competency frameworks, IA risks becoming a guideline-endorsed practice that cannot be delivered with fidelity in routine care, which are key concerns in patient safety and implementation science.^36 38 39^

These barriers to following IA guidelines are significant. However, the study uncovered examples of facilitators that support delivering IA according to guidance. These characteristics are well known within healthcare as support mechanisms and include: leadership engagement driving the culture of the unit,^40 41^ access to knowledge and information supported in mentorship programmes^42^ and peer support models.^43^ These features created micro-environments where IA was valued, supported and integrated into care and are key to sustained implementation.^20^ The learning from this analysis and from the experiences of women^26^ has been used to co-design a quality improvement toolkit to support IA use, which we are evaluating.

## Conclusions

Our study has demonstrated how multilevel factors exert a powerful influence on the implementation of IA in UK maternity care. Yet, where organisational readiness, leadership engagement and supportive conditions are in place, IA can be practised according to guidance. These findings reinforce the importance of aligning policy, infrastructure and culture to sustain evidence-based, woman-centred practices such as IA.

## Data Availability

Data are available upon reasonable request.
