# Supplementary material for: Facilitators and barriers to the practice of intermittent auscultation fetal monitoring in UK maternity services: a qualitative study using the Consolidated Framework for Implementation Research (CFIR)
Source: BMJ Open. 2026 Apr 24;16(4):e115855. doi: 10.1136/bmjopen-2025-115855 (PMC13110571; doi:10.1136/bmjopen-2025-115855)
Supplement: Supplementary file 1 [file bmjopen-2025-115855.reviewer_comments.pdf]

PEER REVIEW HISTORY

BMJ Open publishes all reviews undertaken for accepted manuscripts. Reviewers are asked to complete a checklist review form and are provided with free text boxes to elaborate on their assessment. These free text comments are reproduced below.

ARTICLE DETAILS

Title (Provisional)

Facilitators and barriers to the practice of intermittent auscultation fetal monitoring in UK maternity services: A qualitative study using the Consolidated Framework for Implementation Research (CIFR)

Authors

MacLellan, Jennifer; Ade, Mo; Douthwaite, Megan; Fitzsimons, Bev; Joash, Karen; Mulla, Sumayya; Sanders, Julia; Kenyon, Sara; Pope, Catherine J; Rowe, Rachel

---

VERSION 1 - REVIEW

**Reviewer**	**1**
**Name**	**Fox, Deborah**
**Affiliation**	**University of Technology Sydney**
**Date**	**04-Feb-2026**
**COI**	**None**

Thank you for the opportunity to review this manuscript describing barriers and facilitators to intermittent auscultation in the UK. This is an important topic because midwives in many jurisdictions are becoming deskilled in the use of IA. The dataset is large, providing a rich analysis using the CFIR, which was an appropriate framework to apply to this investigation.

My comments are minor, as follows:

- In Table 1 the bold type in the Constructs column denotes application to IA practice, however, the reader is left wondering why some of the constructs were considered not applicable. I suggest considering a Supplementary file that justifies your choices. For example, under 'Inner setting', 'available resources' is a construct mentioned in the findings but this in not bold in your table. Another example - why are relational connections not included? Relational connections are key to midwifery practice.

- on page 11, you state that IA is 'hard to describe and visualise, rendering the skill invisible'. Please expand and clarify what is meant here.

- there is a typo in the title - 'CIFR' should read 'CFIR'

- Table 3 Midwifery Unit* - I cannot see any reference to the meaning of the *

- please check your spelling of the work practice/practise throughout the manuscript. Practice is a noun and practise is a verb, hence practicing is a misspelling. There are some inconsistencies throughout.

- page 21 line 420 - typo - 'wont' should read 'won't'

- Figure 1 - you refer in the findings to the benefits of understanding fetal physiology. Have you considered if this is a facilitator that belongs in this Figure?

- There are frequent missing full stops - see for example lines 481, 483, 489, 491 and elsewhere

I look forward to seeing this progress to publication, best wishes.

**Reviewer**	**2**
**Name**	**Maude, Robyn**
**Affiliation**	**Victoria University of Wellington, School of Health**
**Date**	**01-Mar-2026**
**COI**	**None**

This is an important study. My passion is teaching and using IA in practice. I understand the considerable pressures from medical colleagues and institutions to push CTG usage, but the evidence for its application for well women with uncomplicated pregnancies is weak. Midwifery researchers need to continue to build the body of evidence around IA. In a world where we are dealing more and more with disasters caused by climate change and civil unrest, midwives MUST learn and maintain the skill of IA, because we cannot always guarantee there will be power to run the CTG. Looking forward to more from your group and perhaps some collaborations.

BMJ Open Review

Facilitators and barriers to the practice of intermittent auscultation fetal monitoring in UK maternity services: A qualitative study using the Consolidated Framework for Implementation Research (CIFR)

Thank you for asking me to review this publication – the topic area strongly resonates with me.

The **title** contains all the information the reader needs to understand the content. The **abstract** is well-constructed and meets all the guidelines for this journal's abstracts.

The **strengths and limitations** are clear. One question I initially had was on page 1, line 31, regarding the number of sites where the study was carried out. The abstract states seven NHS maternity services across England in 2024. However, at line 31, the sample size is stated

as 11 maternity units. It took until page 9, Table 2 Characteristics of study sites, to appreciate that some of the seven maternity services had multiple maternity units.

- To clarify this earlier, I recommend that the authors note 11 maternity units across seven maternity services in the abstract.

The **background** sets the scene well, and the literature reviewed for this section is appropriate. One more discrepancy between the abstract and this section concerns the participants in this study. The abstract on page 1, line 8 states that the participants are midwives and other maternity care professionals, whilst at the end of the background on page 3, lines 72/73, it states "we explore midwifery practice of IA…" So, who are the maternity care professionals involved other than midwives? Table 3 on page 10 describes the characteristics of the participants and shows midwives, student midwives, an obstetrician and a maternity support worker. Why were they included? See also Study Design on page 3, line 76 – "observation of midwives…"

- Please include the research question at the end of this section.
- Clarify who the participants are and align all areas where you talk about midwives, maternity care professionals, maternity practitioners and practitioners.

The **theoretical approach** is well described and very appropriate. Table 1 on pages 5 and 6 maps well against the framework described by Damschroder, Reardon, Opra Widerquist and Lowery, 2022. It is an excellent guide for this study.

The section on **participants and sampling** is thorough and clearly describes the consent process, including consent from the women receiving care from a midwife participant. Again, in this section, we need to describe the participants in accordance with the comments above. You have used 'women' on page 7, line 102. Many journals now have a statement on inclusive language that uses 'pregnant people'. My preference is for additive language such as 'women and pregnant people'.

- Check the journal's author guidelines for preferred language.

The section headed **"Patients and Public Involvement"** needs greater explanation. The two sentences do not do justice to the heading. We need more details about the sense-checking exercise. Please change the word 'Patient' to a term that is more consumer-friendly and reflective of the relational care model of midwifery.

- What was the purpose and validity of this exercise?
- How were the women with lived experience of IA recruited for the workshop?
- Who were the maternity professionals participating in the collaborative workshop?

The **Findings** section begins with Table 2: Characteristics of study sites. It is still not clear where the 11 maternity units from the seven maternity services were included in the study. See the comment above regarding the inclusion of an obstetrician and maternity support worker as participants.

- What does the asterisk on the Maternity Unit in the current area of work relate to?

The **findings** are well presented and well supported by quotes from participants. However, it is unclear where the observation data are provided. I would have preferred to see this

presented explicitly, as there is so much value in seeing the barriers and facilitators through the observers' eyes.

- Change the word "patient" on page 12, Line 209 to "woman" or "women" and "pregnant people".

The **visual summary** is a good addition to the findings, but this section really needed an introduction and discussion of the diagram itself.

The **discussion and conclusion** succinctly summarise the findings and align with current evidence-based literature. It is good to see the research has prompted work on co-designing a quality improvement toolkit to support IA use. Be sure to search widely, including in the Southern hemisphere, for examples of education models and decision-making frameworks for IA.

The **declarations** all appear complete.

References are relevant and well-structured. All references in the list are in the main body of text.

- Check references 5 and 8 – this is a repeated reference

VERSION 1 - AUTHOR RESPONSE

Reviewer: 1
Dr. Deborah Fox, University of Technology Sydney
Comments to the Author:

Reviewer comment	Author response
In Table 1 the bold type in the Constructs column denotes application to IA practice, however, the reader is left wondering why some of the constructs were considered not applicable. I suggest considering a Supplementary file that justifies your choices. For example, under 'Inner setting', 'available resources' is a construct mentioned in the findings but this in not bold in your table. Another example - why are relational connections not included? Relational connections are key to midwifery practice.	*Thank you for this important point. We have added to our explanation in the paper to try and make this clearer. We identified data supporting many of the CFIR constructs in our analysis, and in the paper we have focussed on those expressed most strongly in the data (in the interests of coherence and word count).*<br>*In relation to the 'available resources' construct not being in bold, this was an oversight which we have corrected in the table.*<br>*The relational connections construct in the inner setting domain was interpreted by the research team to be structural rather than interpersonal, hence is not highlighted.*
on page 11, you state that IA is 'hard to describe and visualise, rendering the skill invisible'. Please expand and clarify what is meant here.	*Thank you for this comment. We have expanded this and clarified what we mean here.*
there is a typo in the title - 'CIFR' should read 'CFIR'	*Thank you for noticing the typo, we have now corrected it.*
Table 3 Midwifery Unit* - I cannot see any reference to the meaning of the *	*This is a legacy * and has now been removed*

please check your spelling of the work practice/practise throughout the manuscript. Practice is a noun and practise is a verb, hence practicing is a misspelling. There are some inconsistencies throughout.	*# Thank you for this observation, we have carefully proof read and corrected these spellings.*
page 21 line 420 - typo - 'wont' should read 'won't'	*Corrected*
Figure 1 - you refer in the findings to the benefits of understanding fetal physiology. Have you considered if this is a facilitator that belongs in this Figure?	*Thank you for this recommendation. The figure reflects the shape and content of the Listen2Baby Toolkit. We have removed it from this paper as it represents an extension of the co-design activity. It will be presented in our companion paper describing the co-design of the Listen2Baby Toolkit.*
There are frequent missing full stops - see for example lines 481, 483, 489, 491 and elsewhere	*Thank you for this observation, this has now been corrected.*

Reviewer 2

Reviewer comment	Author response
page 1, line 31, regarding the number of sites where the study was carried out. The abstract states seven NHS maternity services across England in 2024. However, at line 31, the sample size is stated as 11 maternity units. It took until page 9, Table 2 Characteristics of study sites, to appreciate that some of the seven maternity services had multiple maternity units.<br>• To clarify this earlier, **I recommend that the authors note 11 maternity units across seven maternity services in the abstract.**	*Thank you for this recommendation, we have rephrased to 11 maternity units across seven maternity services in the abstract as suggested.*
The abstract on page 1, line 8 states that the participants are midwives and other maternity care professionals, whilst at the end of the background on page 3, lines 72/73, it states "we explore midwifery practice of IA…" So, who are the<br>maternity care professionals involved other than midwives? Table 3 on page 10 describes the characteristics of the participants and shows midwives, student midwives, an obstetrician and a maternity support worker. **Why were they included**?	*IA is predominantly practised by midwives, however obstetricians need to understand this type of monitoring to support its use and understand the findings if an intra-partum transfer is required. In some of the settings, maternity support workers are the second person at birth, they can provide insights into the environment of IA practice.*<br>*We have explained this on page 3:*<br><br>*'We purposively sampled for diverse experiences of and perspectives on IA including from obstetricians and maternity support workers to give a broad holistic view of the context in which IA is practised.'*

See also Study Design on page 3, line 76 – "observation of midwives…"<br>• Please include the research question at the end of this section.<br>• Clarify who the participants are and align all areas where you talk about midwives, maternity care professionals, maternity practitioners and practitioners.	*Thank you for this recommendation, we have included the research question and clarified who the participants are.*
You have used 'women' on page 7, line 102. Many journals now have a statement on inclusive language that uses 'pregnant people'. My preference is for additive language such as 'women and pregnant people'.<br>• Check the journal's author guidelines for preferred language.	*We have included a statement justifying our choice of terminology, thank you for drawing our attention to this.*
The section headed **"Patients and Public Involvement"** needs greater explanation. The two sentences do not do justice to the heading. We need more details about the sense-checking exercise. Please change the word 'Patient' to a term that is more consumer-friendly and reflective of the relational care model of midwifery.<br>• What was the purpose and validity of this exercise?<br>• How were the women with lived experience of IA recruited for the workshop?<br>• Who were the maternity professionals participating in the collaborative workshop?	*Thank you for this feedback, we have included more detail and signposted to the companion paper which is in development describing the co-design process*.
Table 2: Characteristics of study sites. It is still not clear where the 11 maternity units from the seven maternity services were included in the study. What does the asterisk on the Maternity Unit in the current area of work relate to?	*Apologies, this is a legacy * and has now been removed.*
The **findings** are well presented and well supported by quotes from participants. However, it is unclear where the observation data are provided. I would have preferred to see this presented explicitly, as there is so much value in seeing the barriers and facilitators through the observers' eyes.	*Thank you for this comment. We have now more clearly marked which data came from researcher observation.*
Change the word "patient" on page 12, Line 209 to "woman" or "women" and "pregnant people".	*Corrected*
The **visual summary** is a good addition to the findings, but this section really needed an introduction and discussion of the diagram itself.	*Thank you for this recommendation. As we were discussing as a team, we felt the diagram represented an evolved iteration of the baseline CFIR findings following co-design work. The*

	figure will instead feature in our paper on co-design of the Listen2Baby Toolkit.
• Check references 5 and 8 – this is a repeated reference	*Thank you for this observation, we have now removed this duplicate reference.*
